# Investigating the Reasons for Receiving the Second Booster Dose of the COVID-19 Vaccine in Adults and in People with Chronic Medical Conditions in Southern Italy

**DOI:** 10.3390/vaccines11040737

**Published:** 2023-03-27

**Authors:** Grazia Miraglia del Giudice, Lucio Folcarelli, Giorgia Della Polla, Annalisa Napoli, Italo Francesco Angelillo

**Affiliations:** 1 Department of Experimental Medicine, University of Campania “Luigi Vanvitelli”, Via Luciano Armanni 5, 80138 Naples, Italy; 2 Department of Public Health and Laboratory Services, Teaching Hospital, University of Campania “Luigi Vanvitelli”, Via Luciano Armanni 5, 80138 Naples, Italy

**Keywords:** adults, chronic medical conditions, COVID-19, frailty, Italy, second booster dose, vaccination

## Abstract

This cross-sectional survey explored the attitudes and the reasons, as well their associated factors, for receiving the second booster dose of the COVID-19 vaccine among a sample of all old adults and of people with chronic medical conditions attending two randomly selected immunization centers in Naples (Italy). A total of 438 questionnaires were collected. The majority were male (55.1%) and the median age was 71 years. A higher perception of the vaccine’s utility, measured with a 10-point Likert type scale, has been observed among males, individuals with a higher perception that COVID-19 is a severe illness, with a higher self-awareness of being at risk of infection, and with a higher trust in the information received. The most reported reasons for receiving the second booster dose included protection of themselves and of their family members from getting COVID-19, fear of acquiring the disease, and having a physician’s recommendation. Younger participants, married/cohabitant, and with a higher perception that COVID-19 is a severe illness were more likely to have indicated protecting themselves and their family members as reason for receiving the booster dose. Respondents with a chronic medical condition, with a higher perception that COVID-19 is a severe illness, with a lower trust in the information received, and informed by physicians were more likely to have received the vaccine because they perceived of being at risk of getting a severe form of the SARS-CoV-2 infection. Physicians should play a pivotal role in stressing the importance of the second booster dose and in helping individuals to make decisions.

## 1. Introduction

The coronavirus disease 2019 (COVID-19) continues to determine global public health concern with the emergence of the Omicron variant as the dominant strain around the world. As of 7 March 2023,there were more than 755 million confirmed COVID-19 cases and almost 7 million deaths in the world [1], with over 25 million cases and 188,000 deaths in Italy [2]. Although the Omicron variant has raised concern about vaccine efficacy, it has been shown that the fourth BNT162b2 dose is immunogenic, safe, and somewhat efficacious [3,4]. Since 11 July 2022, in Italy, the Ministry of Health has recommended an additional second booster dose of the mRNA COVID-19 vaccines for adults aged 60 and over and for individuals aged 12 and over with high frailty motivated by concomitant/pre-existing conditions who had received the first booster dose or had the last post-booster infection (date of positive test) at least four months (120 days) earlier [5]. It is well known that the achievement of high vaccination coverage is essential for the success of the campaign to reduce the burden of the disease and to control the transmission. Despite this evidence, it is of great concern that as of 12 March 2023, less than one-third of the eligible population for this second booster dose of the COVID-19 vaccine had received it [6].

Understanding the attitudes regarding the second booster dose and the reasons for receiving this dose are crucial for developing specific interventions to increase vaccine uptake and to generate support for health policy makers in the prevention activities. However, no literature is available on this topic. Therefore, to address this gap, this present cross-sectional survey was designed to characterize the attitudes regarding the second booster dose of the COVID-19 vaccine and the reasons for receiving it among a large sample of vaccinated adults and people with chronic medical conditions in Southern Italy, as well as to identify the associated factors.

## 2. Materials and Methods

### 2.1. Study Setting and Sample Recruitment

This survey was conducted as part of a larger project aimed at investigating perceptions and behaviors towards the COVID-19 vaccination among different groups of people in Southern Italy [7,8,9,10,11,12,13,14,15,16] and the Strengthening the Reporting of Observational Studies in Epidemiology (STROBE) guidelines were followed [17]. The survey was carried out between 20 July and 4 August 2022 in two immunization centers randomly selected from the list of those located in the city of Naples, the southern part of Italy. The study population consisted of all subjects aged 60 and over and aged 12 and over with high frailty motivated by concomitant/pre-existing conditions attending on randomly selected days from Monday to Saturday the centers for the administration of the second booster dose of the COVID-19 vaccine. There were no exclusion criteria.

The minimum target sample size of 427participants was determined assuming an expected proportion of 50% of respondents reporting the protection of themselves and their family members from getting COVID-19 as reason for having received the second booster dose of the COVID-19 vaccine, with a margin of error of 5%, considering a confidence interval of 95%, and allowing for an expected response rate of 90%.

### 2.2. Data Collection

Before enrollment, well-trained research investigators, with professional skills in recruiting respondents and knowledge on the topic, approached each potential participant or parent/guardian for those younger than 18 years of age who had been registered for the administration of the second booster dose of the COVID-19 vaccine in the waiting rooms of the centers. The research investigators illustrated for each participant the study objectives and procedures, that the survey was answered on a voluntary basis, that no subject specific identifiers were recorded, that information was confidential, that they had the option to withdraw their participation at any stage without justification, and that by answering the questionnaire they gave the consent to take part in the survey. Informed written consent was obtained from each parent/guardian for the participants younger than 18 years of age. The research investigators asked each participant to complete the questionnaire and to return it immediately once filled. Individuals who had difficulties writing had the option to be interviewed face-to-face by the research investigators. No compensation or incentive was given to the individuals completing the questionnaire.

The Ethics Committee of the Teaching Hospital of the University of Campania “Luigi Vanvitelli” approved the study protocol and the questionnaire.

### 2.3. Questionnaire

The questionnaire was designed based on those used in similar previously published surveys carried out by some of us among a variety of populations [8,9,10,11]. A total of 20 non-selected individuals were interviewed to verify the questionnaire’s clarity, wording, and as well as whether any of the questions were difficult to comprehend. The pilot study considered the psychometric properties of the survey consenting to evaluate whether the questions effectively captured the topic under investigation. The results were not included in the final sample.

The questionnaire, with instructions that aided self-administration by participants, consisted of three sections (Supplementary Material). In the first section, questions were asked about the socio-demographic and anamnestic characteristics (i.e., sex, age, marital status, employment status, educational level, presence of chronic medical condition, having been infected with SARS-CoV-2, and family member/colleague/friend having been infected with SARS-CoV-2). In the second section, questions were asked regarding the attitudes towards the COVID-19 infection (perceived risk of having been infected by SARS-CoV-2 and perceived severity of COVID-19) and the second booster dose of the COVID-19 vaccine (perceived effectiveness, perceived utility, and trust in the information received). These questions were collected on a 10-point Likert type scale, with responses ranging from 1 representing not at all to 10 representing at all. Participants were also asked in a multiple-choice question to indicate the most influential reason(s) for their decision to receive the second booster dose, with 10 options of response and all could be selected. In the third section, the participants were asked to choose from a list of 7 possible options, the sources that they have used to obtain information about the second booster dose, and they were also allowed to indicate additional source(s) not included in the list. Finally, participants were asked whether they needed additional information on this topic.

### 2.4. Statistical Analysis

The following descriptive statistics were computed to summarize the answers of the respondents: frequencies, proportions, means, median, and interquartile range. The chi-square test or Student’s t-test were used to test the association between each variable and the continuous or dichotomous outcome. Variables associated in the bivariate analysis with a *p*-value less than or equal to 0.25 were selected for inclusion into the multivariable analysis. Multivariable linear and logistic regression models with the stepwise variable selection procedure with a threshold of *p* = 0.2 to retain and of *p* = 0.4 to exclude the variables were used to explore the association between several variables and the following outcomes of interest: perception of the utility of the second booster dose of the COVID-19 vaccine (Model 1); protection of themselves and of their family members from getting COVID-19 as reason for having received the second booster dose of the COVID-19 vaccine (Model 2); and perception of being at risk of getting a severe form of SARS-CoV-2 infection as reason for having received the second booster dose of the COVID-19 vaccine (Model 3). The following independent variables have been included in all models: sex (male = 0; female = 1), age (continuous), marital status (unmarried = 0; married/cohabited with a partner = 1), at least one chronic medical condition (no = 0; yes = 1), baccalaureate/graduate degree (no = 0; yes = 1), having been infected by SARS-CoV-2 (no = 0; yes = 1), at least one family member/colleague/friend who has been infected by SARS-CoV-2 (no = 0; yes = 1), level of perception that COVID-19 is a severe illness (continuous), level of trust in the sources of information used about the second booster dose of the COVID-19 vaccine (continuous), self-awareness of being at risk of getting the SARS-CoV-2 infection (continuous), physicians as source of information about the second booster dose of the COVID-19 vaccine (no = 0; yes = 1), and need of additional information about the second booster dose of the COVID-19 vaccine (no = 0; yes = 1). The variable level of the perception of the utility of the second booster dose of the COVID-19 vaccine (continuous) was included in Models 2 and 3.

Odds ratios (OR) with a 95% confidence interval (CI) and standardized regression coefficients (ß) for potential determinants associated with the study outcomes were estimated respectively in the logistic and in the linear regression models. Statistical significance was considered with a two-sided *p*-value equal to or less than 0.05. STATA software version 15.1 was used for performing the statistical analysis.

## 3. Results

### 3.1. Characteristics of the Sample

Of the 452 subjects approached, 438 agreed to participate for an overall response rate of 96.9%. Table 1 displayed the principal characteristics of the study participants. The majority of the participants were male, the median age was 71 years, most were married or cohabited with a partner, 44.1% had at least a university degree, the vast majority were retirees, more than half had at least one chronic medical condition, and 20.1% and 87.2% had a history of SARS-CoV-2 infection in themselves or in at least one family member/colleague/friend.

### 3.2. Attitudes

Survey participants were asked to rate their level of agreement, using a 10-point Likert type scale, with statements regarding attitudes towards the COVID-19 infection and the second booster dose of the COVID-19 vaccine. Regarding the perception that COVID-19 was a serious illness, most respondents (42%) reported the value of 10 with an overall mean value of 8.4. Regarding the self-awareness of being at risk of getting the SARS-CoV-2 infection, the overall mean score was 7.4, and 35.9% of respondents had the higher value of 10. The perception towards the usefulness and utility of the second booster dose was very high with an overall mean value of 8.4 and 8.2, respectively. The final multivariable linear and logistic regression models displayed in Table 2 examined the role of the factors on the different outcomes of interest. In Model 1, the results of the multivariable linear regression analysis indicated that a significantly higher perception of the utility of the second booster dose of the COVID-19 vaccine was observed among males, in individuals who have a higher level of perception that COVID-19 is a severe illness, a higher self-awareness of being at risk of getting the SARS-CoV-2 infection, and a higher level of trust in the sources of information used about the second booster dose. The mean value of respondents’ trust in the sources used to acquire information about the second booster dose was 7.5, but less than one-third (29.5%) expressed higher trust.

### 3.3. Reasons for Receiving the Second Booster Dose of the COVID-19 Vaccine

Table 3 showed the reasons for receiving the second booster dose of the COVID-19 vaccine reported by the study participants. Some of the most reported reasons included protection of themselves (78.1%) and of their family members from getting COVID-19 (42.4%), fear of acquiring the disease (28.1%), having the vaccine recommended by a physician (22.6%), and perception of being at risk of getting a severe form of infection (14.8%). Overall, 38.6% indicated that they want to protect themselves and their family members from getting COVID-19 and the results of the multivariable logistic regression analysis indicated that three variables were significantly associated with this outcome. Younger participants (OR = 0.94; 95% CI = 0.91–0.96), individuals married/cohabitant (OR = 1.75; 95% CI = 1.11–2.73), and with a higher level of perception that COVID-19 is a severe illness (OR = 1.13; 95% CI = 1.01–1.26) were more likely to have indicated protection of themselves and of their family members from getting COVID-19 as reason for having received the second booster dose of the COVID-19 vaccine (Model 2 in Table 2). In the last multivariable logistic regression analysis, four variables resulted significantly associated with the perception of being at risk of getting a severe form of infection as reason for having received the second booster dose of the COVID-19 vaccine. This reason was more likely indicated by respondents with at least a chronic medical condition (OR = 3.24; 95% CI = 1.67–6.25), with a higher level of perception that COVID-19 is a severe illness (OR = 1.35; 95% CI = 1.07–1.71), with a lower level of trust in the sources of information used about the second booster dose of the COVID-19 vaccine (OR = 0.87; 95% CI = 0.78–0.98), and had learned about the second booster dose from physicians (OR = 1.94; 95% CI = 1.07–3.51) (Model 3 in Table 2).

### 3.4. Sources of Information

Most of the sample (91.6%) had learned about the second booster dose of the COVID-19 vaccine. More than half of the respondents indicated mass media (57.1%) as the main source of information. Additional sources were physicians (36.9%) and institutional organizations (24.9%). Only a small number (11.2%) stated that they needed additional information on the second booster dose of the COVID-19 vaccine.

## 4. Discussion

The current findings are important as, to the best of our knowledge, this was the first and largest survey that evaluated vaccinated adults and people with chronic medical conditions about their attitudes regarding the second booster dose of the COVID-19 vaccine and the reasons for receiving it, and quantified the relative contribution of several factors.

The present survey sheds light on the main reasons why adults and people with chronic medical conditions in Southern Italy received the second booster dose of the COVID-19 vaccine. Protection of themselves and of their family members from getting COVID-19, fear of acquiring the disease, having the vaccine recommended by a physician, and the perception of being at risk of getting a severe form of infection were the primary reasons cited by the survey participants. A possible explanation for indicating the protection as the main reason is the extensive and persistent diffusion of SARS-CoV-2 infection that may have contributed to the awareness among the sample with a consequent high sense of urgency regarding this vaccination. Earlier studies in different geographic areas showed comparable results [8,18,19,20]. Furthermore, only 22.6% of the respondents indicated that they were advised by a physician to get this booster dose. The unexpected and surprising finding of the lack of physician’s recommendation is of serious concern given their role in promoting safe and effective care and in counseling about the second booster dose to help the individuals to make informed and useful decisions. This clearly indicated that it is crucial that extensive structured educational and training campaigns need to be carried out on physicians in this country to increase their awareness and for the promotion of the second booster dose to adults and people with chronic medical conditions.

A substantial number of survey participants (91.6%) stated that they had learned about the second booster dose. The most important source of information was the mass media and it is interesting to observe that the physicians ranked second. The heavy use of mass media is an issue of concern. Indeed, even though during the pandemic mass media routinely reported the epidemiological data, some misinformation has been generated especially on the vaccines. It is important to note that physicians as a source of information have a positive significant impact. Indeed, individuals who had received information from a physician were more likely to receive the second booster because they perceive to be at risk of having a severe form of COVID-19 than those who did not use this source. This result highlights that physicians, as all healthcare providers, are a reputable source for disseminating health information and for health promotion and prevention of diseases. They play a pivotal role with a direct impact in helping individuals to acquire and to understand COVID-19-related information and, therefore, in influencing individuals’ decision-making regarding healthcare issues including COVID-19 vaccination. This finding is aligned with the mounting evidence showing that advice or recommendation by physicians is very effective for increasing the level of knowledge, awareness, and vaccination uptake in different groups [8,10,12,21,22,23,24,25,26,27,28]. Moreover, eHealth tools, widely and successfully used during the time of COVID-19 for patients’ management, may be also helpful in immunization programs with education and communication activities on the benefits and to support the promotion of vaccination, and thus with the aims of increasing willingness and uptake.

In addition to the association with the sources of information, the multivariable linear and logistic regression analysis revealed several other significant independent predictors of the three outcomes of interest. It is important to note that among all the socio-demographic and general respondent’s characteristics, age, sex, relationship status, and health status have been identified as important determinants. Males were more likely to have a higher perception of the utility of the second booster dose, whereas young and married/cohabitant respondents were more likely to have received the second booster dose for protection of themselves and of their family members from getting COVID-19 than individuals older and unmarried/not cohabiting. The association with sex may be explained by the number of deaths in Italy, which was higher among males despite the lower numbers of cases compared to females. Regarding the age, younger respondents may be more exposed to SARS-CoV-2 and more worried about the risk of acquiring and transmitting it and, therefore, decide to receive the second booster dose for preventing this infection. Individuals with at least one chronic medical condition were more likely to have received the second booster dose because they perceive to be at more risk of getting a severe form of SARS-CoV-2 infection than healthy individuals. This association may be explained by the fact that they put more emphasis and interest in health issues and thus they are more interested in this vaccination. The associations with socio-demographic and general respondent’s characteristics observed in this survey are consistent with the literature [13,14,15,24,25,26,29,30,31,32,33]. Furthermore, these results indicated that some respondent’s attitudes had an important impact on the outcomes. Indeed, being aware that COVID-19 was a severe disease predicts all three outcomes of interest. This is not surprising, since the individual beliefs about COVID-19 disease surely influence the attitude and the decision to get the vaccine as observed in several previous studies from Western countries [33,34,35,36,37,38,39,40,41,42]. It should be noted that another key finding was the influence of a higher self-awareness of the risk of infection on a higher perception of the utility of the second booster dose [19,23]. Finally, a higher level of trust in the information received was significantly associated with respondent higher perception of the utility of the second booster dose [24,38].

The present survey has some potential methodological limitations that should be considered when interpreting the findings. First, as in all cross-sectional study design, there is no evidence of a temporal relationship between independent variables and outcomes of interest. Second, the survey has been conducted in immunization centers in a single geographic area, thus the findings should be interpreted carefully as they may not be generalized to the overall populations of adults and people with chronic medical conditions in Italy. Third, respondents using self-reporting questionnaires may have introduced response and social desirability bias. However, these types of bias, common disadvantages of this data collection method, have been reduced by using a completely anonymous questionnaire. Although this survey has these limitations, there are several advantages of this type of data collection that should be emphasized, such as the low cost, the rapidity, the easier data collection, and the high response rate. Moreover, this is the first survey about the reasons for receiving the second booster dose of the COVID-19 vaccine in adults and in people with chronic medical conditions in Southern Italy and the results have potential intervention implications for the health policy makers.

## 5. Conclusions

In sum, this survey provides useful information about the second booster dose of the COVID-19 vaccine for adults and for people with chronic medical conditions in Southern Italy and underlines the need for implementation and policymaking in order to expedite the achievement of an optimal coverage rate. Lack of recommendations for receiving the second booster dose from physicians is of great concern, since they should play, as with all public health professionals, a pivotal role in stressing the importance of this vaccination and in advising and helping individuals to make decisions, while self-protection and protection of their family members from infection were the main reasons for this sample to receive the booster dose.

## Figures and Tables

**Table 1:** Main socio-demographic and general characteristics of the sample.

Characteristics	N	%
Age, years	71 (65–77) *	
Sex		
Male	240	55.1
Female	196	44.9
Marital status		
Married/cohabited with a partner	277	66.7
Unmarried	138	33.3
Educational level		
High school degree or less	243	55.9
Baccalaureate/graduate degree	192	44.1
Employment		
Retired	300	68.7
Employed	89	20.4
Unemployed	30	6.9
At least one chronic medical condition		
No	186	42.4
Yes	252	57.6
Having been infected by SARS-CoV-2		
No	350	79.9
Yes	88	20.1
Once	83	94.3
Twice	5	5.7
Time of infection ^+^		
Before vaccination	32	35.6
After the first dose of vaccination	3	3.3
After the second dose of vaccination	7	7.8
After the first booster dose of vaccination	48	53.3
At least one family member/colleague/friend who has been infected by SARS-CoV-2		
No	56	12.8
Yes	382	87.2
Having been vaccinated against influenza in the previous year		
No	162	37
Yes	276	63

Number for each item may not add up to total number of the study population due to missing value. * median (interquartile range). ^+^ of individuals who had been infected twice, two had been infected before the vaccination and after the first booster dose; one before the vaccination and after the first dose; one before the vaccination and after the first booster dose; one after the second dose and after the first booster dose.

**Table 2:** Multivariable linear and logistic regression models for the identification of the predictors for the different outcomes of interest.

Variable	ß Coeff.	SE	*t*	*p*
Model 1. Perception of the utility of the second booster dose of the COVID-19 vaccine F(7, 415) = 27.36, *p* < 0.0001, R^2^ = 31.6%, adjusted R^2^ = 30.4%				
Higher level of trust in the sources of information used about the second booster dose of the COVID-19 vaccine	0.30	0.03	9.33	<0.001
Higher self-awareness of being at risk of getting the SARS-CoV-2 infection	0.14	0.03	4.31	<0.001
Higher level of perception that COVID-19 is a severe illness	0.12	0.04	2.79	0.006
Male	−0.32	0.16	−2.02	0.044
Physicians as source of information for the second booster dose of the COVID-19 vaccine	0.26	0.16	1.60	0.11
At least one family member/colleague/friend who has been infected by SARS-CoV-2	0.31	0.24	1.30	0.195
Older	0.01	0.01	0.97	0.33
	OR	SE	95% CI	*p*
Model 2. Protection of themselves and of their family members from getting COVID-19 as reason for having received the second booster dose of the COVID-19 vaccine Log likelihood = −261.22, χ^2^ = 47.32 (5 df), *p* < 0.0001				
Younger	0.94	0.01	0.91–0.96	<0.001
Married/cohabited with a partner	1.75	0.40	1.11–2.73	0.015
Higher level of perception that COVID-19 is a severe illness	1.13	0.06	1.01–1.26	0.037
Not having a chronic medical condition	0.77	0.16	0.51–1.17	0.229
Need of additional information about the second booster dose of the COVID-19 vaccine	1.44	0.47	0.76–2.73	0.263
Model 3. Perception of being at risk of getting a severe form of SARS-CoV-2 infection as reason for having received the second booster dose of the COVID-19 vaccine Log likelihood = −152.85, χ^2^ = 50.34 (10 df), *p* < 0.0001				
Having at least one chronic medical condition	3.24	1.09	1.67–6.25	<0.001
Higher level of perception that COVID-19 is a severe illness	1.35	0.16	1.07–1.71	0.01
Lower level of trust in the sources of the information used about the second booster dose of the COVID-19 vaccine	0.87	0.05	0.78–0.98	0.022
Physicians as source of information for the second booster dose of the COVID-19 vaccine	1.94	0.59	1.07–3.51	0.029
Younger	0.97	0.01	0.94–1.01	0.072
Female	1.71	0.51	0.94–3.08	0.08
Married/cohabited with a partner	1.68	0.54	0.88–3.17	0.112
High school degree or less	0.61	0.19	0.33–1.12	0.113
At least one relative/colleague/friend who has been infected by SARS-CoV-2	2.18	1.25	0.71–6.71	0.175
Higher self-awareness of being at risk of getting the SARS-CoV-2 infection	1.09	0.73	0.96–1.25	0.186

**Table 3:** Reported reasons for receiving the second booster dose of the COVID-19 vaccine.

	N	%
To protect myself from getting COVID-19	342	78.1
To protect family members from getting COVID-19	186	42.4
Fear of acquiring COVID-19	123	28.1
To protect others from getting COVID-19	100	22.8
Recommended by a physician	99	22.6
COVID-19 is a very contagious disease	95	21.7
COVID-19 is a severe disease	78	17.8
Perception of being at risk of getting a severe form of SARS-CoV-2 infection	65	14.8
Second booster dose efficacy	63	14.3
Second booster dose safety	38	8.6

## Data Availability

The anonymous data presented in this study are available on request from the corresponding author.

## Supplementary Materials

The following supporting information can be downloaded at: https://www.mdpi.com/article/10.3390/vaccines11040737/s1, Supplementary Material: Questionnaire.
