# Supplementary material for: Investigating the Reasons for Receiving the Second Booster Dose of the COVID-19 Vaccine in Adults and in People with Chronic Medical Conditions in Southern Italy
Source: Vaccines (Basel). 2023 Mar 27;11(4):737. doi: 10.3390/vaccines11040737 (PMC10146427; doi:10.3390/vaccines11040737)
Supplement: Supplementary file 1 [file vaccines-11-00737-s001.zip › vaccines-2249093-supplementary.pdf]

Supplementary Material. Questionnaire.

A. SOCIO-DEMOGRAPHIC AND ANAMNESTIC CHARACTERISTICS

This section is designed to gather information about your socio-demographic and anamnestic characteristics

A1. How old were you on your last birthday? ___________

A2. What is your gender? □Male □Female

A3. What is your marital status? □ Married □ Cohabiting □ Other (*please specify*) ___________________

A4. What is your highest educational level? □ None □ Primary school □ High school □ Baccalaureate □ Graduate □ Other (*please specify*) ___________________

A5. What is your occupation? ____________________________

A6. Do you have any chronic medical condition? □No □Yes (*please specify*) ______________________

A7. Have you ever had a test that showed you had been infected by SARS-CoV-2? □No (*go to A8*)

□ Yes, how many times ______

□Before the vaccination: __ times □After the first dose of vaccine: __ times

□After the second dose of vaccine:__ times □After the third dose of vaccine: __ times

A8. Has any family member/colleague/friend been infected by SARS-CoV-2? □No □Yes

A9. Have you been vaccinated against influenza last year? □ No □ Yes

B. ATTITUDES

This section is designed to explore your attitudes towards SARS-CoV-2 infection and its vaccine

B1. On a scale from 1 to 10, how serious do you consider the SARS-CoV-2 infection? *(1 indicates that you do not consider it serious at all, 10 if you consider it a very serious disease)*

Not serious 1 2 3 4 5 6 7 8 9 10 **Very serious**

B2. On a scale from 1 to 10, how much do you perceive yourself at risk of getting the SARS-CoV-2 infection? *(1 indicates that you do not consider yourself at risk at all, 10 if you consider yourself very much at risk)*

Not at risk 1 2 3 4 5 6 7 8 9 10 **Very much at risk**

B3. On a scale from 1 to 10, how useful do you consider the second booster dose of the COVID-19 vaccine? *(1 indicates that you do not consider it useful at all, 10 if you consider it very useful)*

Not useful 1 2 3 4 5 6 7 8 9 10 **Very useful**

B4. On a scale from 1 to 10, how protective for you do you consider the second booster dose of the COVID-19 vaccine? *(1 indicates that you do not consider it protective at all, 10 if you consider it very much protective)*

Not protective 1 2 3 4 5 6 7 8 9 10 **Very much protective**

B5. On a scale from 1 to 10, how much trust do you have in the information you receive on the second booster dose of the COVID-19 vaccine? *(1 indicates that you have no trust, 10 if you have full trust)*

No trust 1 2 3 4 5 6 7 8 9 10 **Full trust**

**B6.** Why did you decide to receive the second booster dose of the COVID-19 vaccine? *(more than one answer is allowed)*	
□ To protect myself from getting COVID-19	□ Fear of acquiring COVID-19
□ To protect my family members from getting COVID-19	□ To protect others from getting COVID-19
□ COVID-19 is a severe disease	□ COVID-19 is a very contagious disease
□ The second booster dose is safe	□ Recommended by a physician
□ The second booster dose is effective	□ Feel to be at risk of getting a severe form of SARS-CoV-2 infection

C. INFORMATION

The section is designed to explore your sources of information about the second booster dose of the COVID-19 vaccine

C1. Which sources did you use to receive information about the second booster dose of the COVID-19 vaccine? (*more than one source is allowed*) □None

□Physicians □Internet □Institutional organizations □Social media (Facebook, Twitter, Instagram, YouTube, etc.) □Mass media □Friends and Family □Scientific journals □ Other (*please specify*) ___________

C2. Do you feel you need additional information on the second booster dose of the COVID-19 vaccine? □No □Yes
